# Impact of *MBL* and *MASP-2* gene polymorphism and its interaction on susceptibility to tuberculosis

**DOI:** 10.1186/s12879-015-0879-y

**Published:** 2015-03-25

**Authors:** Mengshi Chen, Ying Liang, Wufei Li, Mian Wang, Li Hu, Benjamin Kwaku Abuaku, Xin Huang, Hongzhuan Tan, Shi Wu Wen

**Affiliations:** Department of Epidemiology and Health Statistics, School of Public Health, Central South University, Changsha, Hunan 410008 PR China; Hunan Children’s Hospital, Ziyuan RD 86, Changsha, Hunan 410007 PR China; School of Public Health, Xinjiang Medical University, Urumqi, Xijiang 830054 PR China; Department of Nursing, Shaoyang Medical College, Shaoyang, Hunan 422000 PR China; Beijing Center for Diseases Prevention and Control, Beijing, 100013 PR China; Department of Epidemiology, Noguchi Memorial Institute for Medical Research, College of Health Sciences, University of Ghana, PO Box LG581, Legon, Accra, Ghana; Department of Obstetrics & Gynecology, University of Ottawa, The Ottawa Hospital, 501 Smyth Road, Ottawa, Ontario Canada

**Keywords:** Tuberculosis, MBL, MASP-2, Gene, Interaction, RERI

## Abstract

**Background:**

Mannose-binding lectin (MBL) and MBL-associated serine proteases 2 (MASP-2) are important proteins in the lectin pathway of the immune system. Polymorphism of *MBL* and *MASP-2* genes may affect the serum concentration of MBL and MASP-2. This study explores the association between *MBL* and *MASP-2* gene polymorphism and their interactions and the susceptibility to tuberculosis (TB).

**Method:**

A total of 503 patients with TB and 419 healthy controls were recruited to participate in this case-control study. PCR-SSP technology was applied to genotype rs7096206 of *MBL* genes and rs2273346 and rs6695096 of *MASP-2* genes. Demographic data and some exposure information were also obtained from study participants. Unconditional logistic regression analysis was used to identify association between the various factors and TB whilst Marginal Structural Linear Odds Models were used to estimate the interactions.

**Results:**

Both genotype GC at rs7096206 of *MBL* genes and genotype TC at rs2273346 and rs6695096 of *MASP-2* genes were more prevalent in the TB patient group than the healthy control group (*P* < 0.05, OR 1.393, 1.302 and 1.426 respectively). The relative excess risk of interaction (RERI) between rs7096206 of *MBL* genes and rs2273346 and rs6695096 of *MASP-2* genes was 0.897 (95% CI: 0.282, 1.513) and 1.142 (95% CI: 0.755, 1.530) respectively (*P* < 0.05).

**Conclusion:**

Polymorphisms of *MBL* (rs7096206) and *MASP-2* (rs2273346 and rs6695096) were associated with the susceptibility of TB, and there were gene-gene interactions among them.

## Background

Tuberculosis (TB) is a global public health issue posing serious harm to human health. China has the second highest TB burden in the world. According to the fifth TB epidemiological sampling survey in China in 2010 [1], TB prevalence in China was 459/100,000 among people aged 15 years or older. It has been estimated that [2] one-third of the world’s population is infected with tubercle bacilli whilst only 10% of people infected with *Mycobacterium tuberculosis* become TB patients, indicating remarkable individual differences which may be related to nutrition, constitution [3], specific and non-specific resistance [4,5] and genetic susceptibility [6,7].

According to research findings, after the pathogenic microorganism invades the body, mannose-binding lectin (MBL) binds with mannan residues on its surface, and activate MBL-associated serine Proteases (MASPs) and the lectin pathway of the complement system, generating non-specific immune responses [8,9]. Protein MASP-2 and MAp19 encoded by *MASP-2* genes both can bind with MBL, generate MBL-MASP compound, and then activate C4-C9 components in the complement system, generating membrane attack complex and opsonin as well as other inflammatory cytokines promoting the killing of pathogenic microorganisms [10,11].

Previous studies suggest that polymorphisms of *MBL* genes in the promoter region and structural region affect the formation of MBL multimer and serum MBL concentration. The reduction of MBL multimer results in the impaired binding with ligand and the increased likelihood of being degraded by metalloproteinase [12-14]. *MASP-2* gene mutation also facilitates the changes in the serum concentration of the proteins it encodes (namely, MASP2 and Map19), and results in the impaired binding with MBL and ficolin molecules, consequently blocking activation of the lectin-complement pathway resulting in impaired functioning of the non-specific body immune system [15,16].

The impact of *MBL* gene polymorphism on susceptibility to TB has been reported in different regions and among different races [17-19], though the findings are inconsistent between studies. Liu W and colleagues conducted analysis of the six polymorphic sites (A/B, A/C, A/D, H/L, Y/X and P/Q) of *MBL* genes in the Chinese Han population (including 152 male TB patients and 293 healthy males as controls), and found that only the site H/L was associated with susceptibility to TB [20]. Contrary to this, SHI J and colleagues reported that A/B was associated with susceptibility to TB in the Han population, whilst P/Q was not [21]. Further to these inconsistencies, Soborg and Selvaraj reported that low serum MBL levels could reduce tubercle bacilli infections [18,19], whilst other studies found that high of serum MBL levels [22,23] could reduce *tubercle bacilli* infections. Although studies have shown that Promoter -221(Y/X) mutation (YX, XX) [22] and HYB haplotype [23] could lead to decreased serum MBL [12,24] and enhanced susceptibility to TB. Liu Wei and colleagues reported that [20] the promoter -221(Y/X) mutation in the Han population was not associated with susceptibility to TB. No statistically significant difference was observed possibly due to the small sample size, and therefore the need for large sample sizes to explore the association between Y/X (rs7096206) and the susceptibility to TB in the Han population. The polymorphism of *MASP-2* genes was also found to be associated with serum protein levels [15,25,26] and susceptibility to multiple diseases [27-29]. If rs2273346 (p.V377A) mutation (TC, CC) can lead to decreased serum MASP-2 concentration, is the mutation associated with susceptibility to TB? The site of rs6695096 is located at intron 7 and it is not clear that the polymorphism of this locus is associated with the susceptibility to TB. This case-control study explores the polymorphism of the rs7096206 of *MBL* and the rs2273346 and rs6695096 of *MASP2* genes in the Han population in Hunan Province, China, as well as their gene-gene interactions, in order to determine their impact on the susceptibility to TB.

## Methods

### Ethical issues

The study protocol was approved by Ethical Review Committee of the Central South University Ethics Review Committee (*XYSM HSP#: 2007122002A*). All subjects enrolled in this study were over 19 years old and so parental consent was not required. Written informed consent was obtained from all subjects according to guidelines from the ethical review committee.

### Sources of cases

Stratified sampling method was used to randomly select four county-level CDCs (i.e. Qidong County CDC, Yueyanglou District CDC, Yueyang County CDC and Hongjiang City CDC) from a total of 122 in Hunan Province. This was followed by the random selection of new TB cases registered by the four CDCs in 2009. All cases were TB patients confirmed with the TB diagnosis criteria [30] developed by Chinese Ministry of Health.

### Sources of healthy controls

Stratified sampling method was used to randomly select one community health service center (i.e. Xingang Community Health Service Center) from 14 in the Kaifu district in Changsha city. This was followed by the random selection of one community (Xin’ansi) from six communities covered by the Xingang Community Health Center. Because the ratio of male to female TB patients was about 2.5:1 in Hunan [31], the healthy controls were selected from permanent residents in Xin’ansi Community by a gender-age frequency matching method. All healthy controls were confirmed with a history of contact with *Mycobacterium Tuberculosis*. For healthy controls with BCG scar, the average diameter of PPD (purified protein derivative) induration was ≥10 mm whilst for those without BCG scar and no history of BCG vaccination, the average diameter of PPD induration was ≥5 mm. No abnormalities were found in their chest X-rays.

Both cases and controls were selected from closed Han populations within Hunan province, minority individuals have been excluded in this research.

### Estimation of sample size

Sample size estimation was based on an estimated rs6695096 frequency of 14%; OR = 1.8, *α* = 0.05 (paired) and *β* = 0.10. Based on the above assumptions, 419 subjects were to be selected as cases and healthy controls.

### Information and sample collection

After each subject signed the written informed consent form, a self-administered questionnaire was used to collect demographic and selected information, which included sex, age, marital status, educational background, BMI, smoking status, alcohol drinking, tea drinking, and exposure to kitchen fumes.

5 ml of venous blood from each participant was aseptically collected in EDTA anticoagulant tubes and stored in a 4°C refrigerator before use. A blood DNA kit provided by Shanghai Sangon Biotech Co., Ltd. was then used to extract peripheral white blood cell genome.

### Genotyping

In this study, PCR-SSP technology was used to sequence to genotype the rs7096206 of *MBL* genes and the rs2273346 and rs6695096 of *MASP-2* genes. The site sequence of rs7096206 of *MBL* genes and rs2273346 and rs6695096 of *MASP-2* genes was identified in the Gene bank, and appropriate primers were designed by using Primer Premer5.0, the specificity of which was verified by using Blast software of NCBI. All the primers were produced by Shanghai Sangon Biotech Co., Ltd (Table 1). The PCR reaction system was 20 ul, including 10 ul mixture, 0.8 ul gDNA (10 ng/ul), 0.4 ul upstream primers, 0.4ul downstream primers, and 8.4 ul ddH_2_O. The reaction condition was 94°C, 3 min for 40 cycles (94°C for 30 sec, 58°C for 30 sec, and 72°C for 60 sec), and 72°C extension for 5 min.

**Table 1:** **Primers site sequence of** ***MBL*** **and** ***MASP-2*** **genes**

**Mutants**	**Sense primer**	**Anti-sense primer**	**Enzyme**
rs7096206	5′ TGGGTTGGTGACTAAGGT 3′	5′GGTAGGCACTATGATGAGC 3′	Btg I
rs2273346	5′CAGTAGCAGCAGAGGGAG 3′	5′ CCAGGAGTGTCGGGATTA 3′	Sfc I
rs6695096	5′ TCTGTAAACTGCCTGTCC 3′	5′ ACTACTCCGTAATCCAAG 3′	HpyCH4 III

The enzyme digestion reaction system was 10 ul, including 2 ul PCR product, 1 ul 10X buffer, 0.5 ul corresponding restriction endonuclease, and 6.5 ul ddH_2_O. It was kept at 37°C over night. 5 ul enzyme-digested product was applied to 3% agarose gel (containing 0.5 ug/ml ethidium bromide). Electrolytic buffer solution: 0.5xTBE solution; voltage for sample application: 120 V; electrophoresis: 40 min. Gel imaging processing system was used to observe the electrophoresis results, determine the genotype, and take photos.

### Statistical analysis

Epidata3.0 was used to input data, and SAS9.2 was used to analyze the data. *χ*^2^ test was conducted for the comparison of grouped data and Hardy-Weinberg equilibrium detection. Linkage disequilibrium analysis was evaluated by SHEsis online software (http://analysis.bio-x.cn/myAnalysis.php). The risk associated with individual alleles was calculated as the odds ratio with 95% confidence interval. To exclude possible confounding risk factors, the occurrence of TB was used as the dependent variable, rs7096206 of *MBL* genes and rs2273346 and rs6695096 of *MASP-2* genes were used as the independent variables, and the sex, age, marital status, educational background, BMI, smoking status, alcohol drinking, tea drinking, and exposure to kitchen fumes were used as the covariates, and multivariate unconditional logistic regression analysis conducted. Marginal Structural Linear Odds Models [32] were used for point estimation and interval estimation of the relative excess risk of interaction (RERI). RERI > 0 suggests positive interactions.

## Results

The study participants include 503 TB patients and 419 healthy controls. The TB patient group and the healthy control group exhibited no statistical significance difference (*P* > 0.05) in terms of sex, age, education background and alcohol drinking. Differences in marital status, BMI, tea drinking, smoking status, history of BCG vaccination and exposure to kitchen fumes was statistically significant (*P* < 0.05).

The univariate analysis showed that genotype GC at rs7096206 and genotype TC at rs6695096 were closely associated with TB incidence (OR reaching 1.338 and 1.468, respectively). Multivariate unconditional logistic regression analysis showed that rs7096206 of *MBL* genes and rs2273346 and rs6695096 of *MASP-2* genes were associated with susceptibility to TB. Both genotype GC at rs7096206 of *MBL* genes and genotype TC at rs2273346 and rs6695096 of *MASP-2* genes were more prevalent in the TB patient group than those in the healthy control group (*P* < 0.05), with OR 1.393, 1.302 and 1.426 respectively (Table 2). Linkage disequilibrium analysis showed linkage equilibrium for rs2273346 and rs6695096 of *MASP-2* genes (For controls, *D’* = 0.029, *r*^*2*^ = 0.01; for cases, *D’* = 0.013, *r*^*2*^ = 0.00).

**Table 2:** ***MBL*** **and** ***MASP-2*** **gene polymorphism versus TB incidence**

		**TB patients**		**Healthy controls**		**OR**_**c**_ **(95% CI)**	**OR**_**ad**_ **(95% CI)** ^**#**^
		***N***	**%**	***N***	**%**		
rs7096206	*CC*	325	64.61	296	70.64	1	1
	*GC*	166	33.00	113	26.97	1.338 (1.005,1.781)*	1.393 (1.042,1.861)*
	*GG*	12	2.39	10	2.39	1.093 (0.465,2.567)	1.155 (0.489,2.727)
rs2273346	*TT*	321	63.82	281	67.06	1	1
	*TC*	162	32.21	115	27.45	1.233 (0.925,1.644)	1.302 (1.013,1.784)*
	*CC*	20	3.98	23	5.49	0.761 (0.409,1.415)	0.831 (0.434,1.590)
rs6695096	*TT*	301	59.84	286	68.26	1	1
	*TC*	187	37.18	121	28.88	1.468 (1.110,1.943)*	1.426 (1.062,1.915)*
	*CC*	15	2.98	12	2.86	1.188 (0.547,2.581)	1.358 (0.605,3.048)

^#^Multivariate Logistic regression model was used to adjust the covariates of sex, age, marital status, educational background, BMI, smoking status, alcohol drinking, tea drinking, BCG vaccination, and exposure to kitchen fumes.

For Hardy-Weinberg equilibrium detection, P > 0.05.

*P < 0.05.

Marginal Structural Linear Odds Models were used to analyze the impact of the interactions between *MBL* genes and *MASP-2* genes on susceptibility to TB. Adjusting for the covariates of sex, age, marital status, educational background, BMI, smoking status, alcohol drinking, tea drinking, history of BCG vaccination, and exposure to kitchen fumes, the relative excess risk of interaction (RERI) between rs7096206 of *MBL* genes and rs2273346 and rs6695096 of *MASP-2* genes was found to be 0.897(95% CI: 0.282,1.513) and 1.142(95% CI: 0.755,1.530) respectively (*P* < 0.05), which suggests positive interactions (Tables 3 and 4).

**Table 3:** **Impact of interactions between rs7096206 and rs2273346 on incidence of TB**

		**rs2273346**		**RERI**_**c**_	**RERI**_**ad**_ **(95% CI)**
		***TT***	***TC + CC***		
rs7096206	*CC*	1	1.131	0.566	0.897 (0.282,1.513)*
	*GC + GG*	1.261	1.958		

*P < 0.05, RERI > 0 suggests positive interactions.

**Table 4:** **Impact of interactions between rs7096206 and rs6695096 on incidence of TB**

		**rs6695096**		**RERI**_**c**_	**RERI**_**ad **_ **(95% CI)**
		***TT***	***TC + CC***		
rs7096206	*CC*	1	1.308	0.881	1.142 (0.755,1.530)*
	*GC + GG*	1.190	2.380		

*P < 0.05, RERI > 0 suggests positive interactions.

## Discussion

The incidence of TB is a result of the interactions between *Mycobacterium tuberculosis* and hosts. TB infection and subsequent incidence are affected by many factors, including the odds of exposure to *Mycobacterium tuberculosis*, toxicity of pathogenic bacteria, and the immune function of the host. Previous studies on incidence of TB primarily focused on the tubercle bacilli and impact of environmental risk factors. Over the past years, the impact of host susceptibility genes on disease has been increasingly recognized along with the development of genetic epidemiology.

Recent studies on the association between *MBL* genes and TB have produced different and even contradictory results. Some studies indicated that the mutation of promoter and exon 1 in *MBL* genes may lead to the decline of serum MBL level, while a lower serum MBL level can reduce *tubercle bacilli* infections [18,19]. Some other studies indicated that higher serum MBL levels can reduce tubercle bacilli infections, while higher serum MBL levels are associated with wild-type *MBL* genes [22,33]. Meta-analysis [34] by Denholm and colleagues indicates that polymorphism of *MBL* genes may be associated with serum MBL level rather than susceptibility to TB. These studies ignored the interactions between genes, as well as certain genetic and environmental factors in the analysis of gene and susceptibility to TB. Moreover, small sample sizes cannot detect real association between *MBL* genes and TB. Our study included some possible covariates in the logistic regression model for analyzing the relationships between *MBL* genes and TB, such as sex, age, marital status, educational background, BMI, smoking status, alcohol drinking, tea drinking, BCG vaccination and exposure to kitchen fumes, excluding possible confounding caused by these factors. In this way, our study results are closer to the real situation.

Our study revealed that the promoter -221(Y/X, rs7096206) mutation of the *MBL* genes is associated with susceptibility to TB, and the TB risk of heterozygote GC(YX) is higher than that of wild-type homozygous CC(YY) (OR = 1.393, *P* < 0.05), which are consistent with the findings of a Brazilian study [22]. *MBL* genes are associated with the serum MBL level, and promoter -221(Y/X) mutation (YX, XX) can lead to decreased serum MBL [12,24] and consequently increased susceptibility to TB.

To our knowledge, reports have been made on the association of *MASP-2* genes on other disease conditions but not TB. According to Boldt and colleagues [27], p.D371Y and p.V377A (TC, CC) mutation are associated with the serum MASP-2 level in Chagas patients; Sorensen and colleagues found through twin analysis that genetic inheritance may affect the activity of MASP-2 [26]; Thiel and colleagues found that p.V377A(rs2273346*)* mutation (TC, CC) may lead to decreased serum MASP-2 concentration [15], and when both p.D120G and p.156_159CHNPdup undergo mutation, MASP-2 may undergo misfolding and cannot bind with MBL [16]; Yan Wang and colleagues found that the polymorphism of rs2273346 of *MASP-2* genes is not associated with SARS coronavirus infections in Beijing and Guangzhou [35]; Tulio and colleagues found [29] that hepatitis C virus infection is associated with the polymorphism of *MASP-2* gene p.D371Y(rs12711521*)* but not associated with the polymorphism of p.V377A (rs2273346*)*. Our study reports on the association between *MASP-2* gene polymorphism and TB susceptibility. The study has shown that that rs2273346 and rs6695096 of *MASP-2* genes can increase susceptibility to TB, possibly because the mutation of rs2273346 (p.V377A) (TC, CC) and rs6695096 (TC, CC) can lead to decreased serum MASP-2 concentration and possibly decreased activity of MASP-2, and subsequently impairing the body immune function and increasing the risk of TB. The site of rs6695096 is located at intron7, the mutation of which may affect gene regulation and selective splicing regulation although it will not affect the sequence of amino acids [36,37] and subsequently affect serum concentration and activity of MASP-2. The biological mechanism is yet to be further examined.

Marginal Structural Linear Odds Models analysis showed that RERI between rs7096206 of *MBL* genes and rs2273346 of *MASP-2* genes was 0.8977(95% CI:0.2821, 1.5133), and RERI between rs7096206 of *MBL* genes and rs6695096 was 1.1429(95% CI:0.7556,1.5301). There were significant positive interactions between rs7096206 of *MBL* and both rs2273346 and rs6695096 of *MASP-2*, which suggest that the mutations of both *MBL* genes and *MASP-2* genes can lead to an increased risk of TB. These findings provide important reference information for studies on MBL and MASP-2 interaction mechanism.

There are some limitations to our study. First, there are six known polymorphisms within *MBL-2* that affect the amounts of MBL in human plasma, but we only detected the polymorphism of rs7096206. We could therefore not analyze the impact of other polymorphisms and haplotypes of *MBL* gene on TB susceptibility. Secondly, we did not test for the association between rs6695096 and MASP-2, and did not find any laboratory evidence in literature to support such association. Thirdly, cases and controls in our study were sampled from different regions. However, all the participants have been limited to Han Chinese, and all the possible impacts of non-genetic factors such as sex, age, marital status, educational background, BMI, smoking status, alcohol drinking, tea drinking, and so on, were adjusted. So the results observed in our study should be reliable.

## Conclusion

Polymorphisms of *MBL* (rs7096206) and *MASP-2* (rs2273346 and rs6695096) were associated with TB susceptibility, and there were gene-gene interactions among them. This finding is not only significant for understanding the pathogenesis of TB, but also important for identifying populations at high risk of TB, and developing appropriate population-specific prevention measures to control the spread of TB.
